# Performance of the Malay Audit of Diabetes Dependent Quality of Life-18 and Associates of Quality of Life among Patients with Type 2 Diabetes Mellitus from Major Ethnic Groups of Malaysia

**DOI:** 10.1371/journal.pone.0163701

**Published:** 2016-10-03

**Authors:** Aqil M. Daher, Syed Hassan A. AlMashoor, Than Winn

**Affiliations:** 1 Department of Community Medicine, Faculty of Medicine and Defence Health, National Defence University of Malaysia, Sungai Besi, Kuala Lumpur, Malaysia; 2 Department of Psychiatry, Faculty of Medicine, Universiti Teknologi MARA, Batu Caves, Selangor, Malaysia; 3 Department of Community Medicine, Faculty of Medicine, MAHSA University, Kuala Lumpur, Malaysia; TNO, NETHERLANDS

## Abstract

**Introduction:**

Diabetes Mellitus (DM) is notorious for its metabolic effect, acute and chronic complications and impact on Quality of Life (QoL). Successful intervention to improve QoL necessitates a valid and reliable measurement tool to identify areas of concern to patients with diabetes.

**Objectives:**

To (1) assess the factor structure of the Malay Audit of Diabetes Dependent Quality of Life-18 (ADDQoL-18) questionnaire; (2) determine the impact of DM on QoL; and (3) identify areas of concern to patients with type 2 DM from three major ethnic groups in Malaysia.

**Methods:**

Data was obtained from a cross sectional study involving 256 patients with type 2 DM attending the diabetes clinic of the National University of Malaysia Medical Centre. The Malay version of ADDQoL-18 survey was translated from its English version according to standard guidelines and administered by a trained research assistant. Exploratory Factor Analysis (EFA) with oblimin rotation was used to determine factor structure of the data. Confirmatory Factor Analysis (CFA) was used to confirm the factor structure. Hierarchical liner regression was used to determine factors associated with QoL.

**Results:**

Unforced factor solution yielded two factors for the whole sample. Forced one factor solution was ascertained for the whole sample and for each ethnic group. Loadings ranged between 0.588 and 0.949. Reliability coefficients were all higher than 0.955. CFA showed that the two factor model had better fit statistics. QoL was associated with the use of insulin and desired glycaemic control, longer diabetes duration, worry about diabetes, and diabetes complications.

**Conclusions:**

The Malay ADDQoL-18 is a valid tool to be used among patients with diabetes from different ethnic groups in Malaysia. The use of insulin to achieve desired glycaemic control had more negative impact on QoL than the use of tablets and/or dietary changes.

## Introduction

The prevalence of DM is increasing all over the world and reached an alarming figure by 2013 with an excess of 8.3% of the global population or approximately 382 million people afflicted [1]. This is projected to rise to upwards of 552 million (9.9%) by 2030 [2]. The prevalence of DM among the world’s countries shows a wide range from as low as 4.6% in Yemen [3] to 9.3% of the total US population [4] to as high as 10.7% in the United Arab Emirates [5] and Malaysia (15.2%) [6]. Regionally, Africa was found to have the lowest rate (2.4%), while the increasing prevalence of DM is anticipated for South East Asian countries and for Sub-Saharan Africa. This rise is mainly attributable to urbanization and westernization coupled with genetic predispositions. The prevalence of diabetes in Malaysia has dramatically increased over the last twenty years. A prevalence of 15.2% in 2011 is twice that of 1996 (6.3%). There is also a noticeable difference with regards to race: patients of Indian parentage incur the highest prevalence (19.9%), followed by Malays (11.9%). The lowest rate was found among Chinese (11.4%), but in a global perspective is still comparatively high [7].

DM is a chronic disease for which management is aimed to minimize complications, decrease suffering and improve QoL. Quality of Life (QOL) has emerged as a new paradigm in the assessment of healthcare outcomes and disease burden. QOL is a multi-dimensional concept that encompasses a wide range of attributes affecting individual perception and satisfaction with life. Assessment of QoL among patients with diabetes has taken different approaches. Generic and disease-specific instruments were used previously [8]. ADDQoL is one of the commonly used questionnaires in assessing QoL among patients with diabetes and is proven to be valid and reliable. The survey demonstrated better sensitivity in characterizing disease severity and in detecting areas of major concerns to the patient and/or families and their care givers. Watkins and Connell [9] pointed out that ADDQoL is unique in many aspects. The instrument allows the patient to rate their QoL as if they did not have diabetes, allowing them to imagine their healthy life. Secondly, patients are given the liberty to answer questions deemed to be relevant to each individual patient, thus allowing freedom in answering the questionnaire.

Translated questionnaires may behave differently due to cultural and linguistic differences [10].The factor structure of ADDQoL has been shown to be stable in all studies whether in the original language or when translated. However, it was observed that two factors emerged with EFA in Bradley’s development study [11] and other studies [12–15]; three factors among Slovak population [16] and 4 factors among the British seniors [17]. Nonetheless, a forced one-factor solution has been always opted as the optimum solution in these studies.

Malaysia is a multiracial country adopting the Malay language as its official language. Nonetheless, in view of its multiethnic population, various other languages are common in everyday parlance. Although translation from English to the Malay language increased in the last 25 years, difficulties in translating English terms into Malay has been acknowledged since 1960, as the Malay language vocabulary is highly influenced by firstly Sanskrit, secondly Arabic and thirdly the English language [18].

The Malay version of ADDQoL-18 was translated and validated among a grouped of patients with type 2 diabetes from the Malay race only [13]. We felt the necessity to test the impact of ethnicity on the factor structure of Malay ADDQoL-18 among the three main ethnic groups in Malaysia: Malay, Chinese and Indian, and to assess QoL among this cohort of patients with diabetes.

## Materials and Methods

The data were collected by a trained Research Assistant (RA) from 256 patients with type 2 diabetes attending the diabetes clinic of the National University of Malaysia Medical Centre. Participants were selected through systematic random sampling for a period of six months. As the sampling interval is determined by total number of population and the required sample, it was decided to target 10 patients per day out of those attending the clinic due to the presence of other competing studies in the same centre. Allowing for the possibility of being attached to another study, the sampling interval was minimally varied during the clinic session: Selecting every 7^th^ or 8^th^ patient from the list of patients who presented at the clinic.

The RA contacted each selected patient whilst waiting to see the doctor, explained to him/her the rationale of the study, verified whether the patient can read and understand the language of the questionnaire, and subsequently obtained written consent from participant. Eligible patients were handed a self-administered questionnaire to be completed and returned on the spot. Inclusion criteria were: (1) A history of type 2 DM for more than one year; (2) Malaysian citizen able to read and write in Malay language; and (3) Stable diabetes without need for hospital admission in the last 3 months prior to this study. The study was approved by the ethics committee of National University of Malaysia and Ministry of health.

### Instrument

The ADDQoL-18 is composed of two overview items and18 life domains. The overview items are the Present QoL (PQoL) and the Diabetes Impact on QoL (DMQoL). Applicable life domains are rated for impact of diabetes and for importance to quality of life. These are multiplied together to produce a weighted impact score for each domain. Applicable domains are averaged into a single score: Average Weighted Impact (AWI). The Malay version of ADDQoL-18 was translated from the English version of ADDQoL-18 according to standard guidelines and included two forward and two backward translators [13]. Two native Malay speakers who were also fluent in English carried out the translation to the target language while two English speakers who were expert in Malay language performed the backward translation to the original language. Discrepancies were resolved and the harmonized Malay version of ADDQoL was pretested among a sample of diabetic patients. The final Malay version was tested for its factor structure and reliability among a sample of diabetic patients in northern Malaysia. Data about socio-demographic factors, disease factors (complications, glycaemic control, diabetes duration, treatment modality), and satisfaction with waiting time were collected as predicators of QoL.

### Statistical Analyses

Descriptive statistics were produced for the sample. Evidence of correlation between items was examined through a correlation matrix: Kaiser-Meyer-Olkin (KMO) criteria and Bartlett’s test. A value of 0.7 or above for KMO [19], and a significant p value of Bartlett’s test indicated sufficient correlation among items.

Principal component analysis (PCA) with oblimin rotation was used to identify the factor structure that accounts for the highest percentage of explained variance. Number of factors was based on eigenvalue more than 1.The pattern matrix was then examined for loadings and cross loading. An item loading of more than 0.4 was considered significant and to be retained provided that it did not load highly to other factors. Item discriminant validity was ascertained with “the finding of high correlation between the item and its hypothesized a construct than the correlation with other construct”. A difference of more than 0.15 in the cross-loadings was considered supportive of item discriminant validity [20]. CFA was used to confirm the factor structure among the whole sample.

Putative variables, including socio-demographic characteristics, were tested separately in a regression model. Interaction (moderating effect) was tested between variables that were hypothesized to interact. All variables were then included in a hierarchical linear regression model to identify factors associated with QoL. According to hierarchical linear regression algorithm, the independent variables are entered cumulatively according to some specified hierarchy which is dictated in advance by the purpose and logic of the research [21]. Independent variables in this study were grouped into blocks and entered into model in sequence from 1^st^ to 4^th^ block. The first block included sociodemographic variables; the second block included other predictors associated with diabetes QoL; the third block included the moderator variable under investigation; and the last block included interaction term.

## Results

### Descriptive statistics of the sample

Table 1 shows the characteristics of the study sample. Among the 335 targeted patients, two hundred and fifty six consented and returned the completed questionnaire yielding a response rate of 76.4%. Respondent age ranged between 30 and 80 years with a mean (± SD) of 56.79 (± 10.5) years. Out of the total respondents, 58.6% were females. There were 48.4% Malays, 26.6% Chinese and 25% Indians. The majority of participants reported having completed secondary education (47%), were married (88.1%), and were employed at the time of the study (38.9%). Approximately 63.7% of respondents were on a diet or taking oral modes of treatment; the rest were using insulin. More than a third of the respondents (39.8%) have had diabetes for more than 10 years and 67.5% had a level of HbA1c greater than 48 mmol/mol (6.5%).

**Table 1:** Characteristics of study sample.

Variable		n	%
Gender	Male	106	41.4
	Female	150	58.6
Race	Malay	124	48.4
	Chinese	68	26.6
	Indian	64	25
Educational Level	No formal education	13	5.1
	Primary	63	24.8
	Secondary	121	47.6
	Diploma/University	57	22.5
Marital Status	Single	6	2.4
	Married	222	88.1
	Separated/Divorced/Widow	24	9.5
Occupation	Unemployed	61	24.2
	Employed	98	38.9
	Pensioner	55	21.8
	Housewife	38	15.1
Treatment	Diet/Oral	158	63.7
	Insulin	90	36.3
Duration of Diabetes	< 10 yrs	136	60.2
	≥ 10 yrs	90	39.8
Glycemic Control (HbA1c)	< 48mmol/mol (6.5%)	79	32.5
	≥ 48mmol/mol (6.5%)	164	67.5
Diabetes Worry		205	82.7
Retinopathy		103	41.5
Neuropathy		86	34.3
Foot ulcer		35	13.8
Renal Complications		34	13.5
Ischemic Heart Disease		28	11.1
Cardiovascular accident		13	5.2
Age Mean(SD)		56.85(± 10.5)	
BMI		27.69 (± 5.22)	
Systolic Blood Pressure		137.71(± 1.11)	
Diastolic Blood Pressure		76.64 (± 0.75)	

In terms of diabetes related worry, it was found that 82.7% of the patients were worried about diabetes complications. With regard to any complications the patients had, retinopathy and neuropathy were the most commonly reported complications among the respondents. Foot ulcer and renal complications were almost equally reported. Only 11.1% had ischemic heart diseases while Cardiovascular Accident (CVA) was reported in fewer than 6% of the patients.

### Factor structure and reliability

KMO was found to be 0.943 and Bartlett's test of sphericity was significant with p = <0.001, which supports factor analysis. The PCA with oblimin rotation for the whole sample (Table 2) showed a two factors solution. The first factor included the first 15 life domains and the three domains of freedom to eat, enjoyment of food and freedom to drink loaded highly into the second factor. When these two factors were forced, loadings ranged between 0.657 and 0.857. In terms of ethnic groups, there was a similar unforced factor structure among Malay respondents where the last three domains loaded highly into a second factor. However, the loadings of forced one factor were all higher than 0.593. In regards to the Chinese group, three factors were extracted with no clear pattern of loadings. A forced one factor showed loadings higher than 0.588. In relation to Indian participants, only one factor extracted with loadings ranging between 0.728 and 0.949. All factor solutions explained more than 50% of the variance (Malay 57.80%, Chinese 62.38% and Indians 72.50%), with the forced factor solution of the whole sample explained (61.42%). Cronbach’s alpha reliability coefficient was high for all ethnic groups: higher than 0.955. Item total correlation corrected for overlap was above 0.4. None of the item deletions performed would increase the reliability.

**Table 2:** Items loading of the Malay ADDQoL-18.

	Malay			Chinese				Indian	Whole Sample		
	Unforced		forced	Unforced			forced		Unforced		forced
	1	2	1	1	2	3	1	1	1	2	1
working life	**.663**	.106	**0.714**	.612	.120	**.405**	**0.677**	**0.846**	**0.761**	-0.017	**0.729**
family life	**.577**	.294	**0.754**	.421	-.002	**.603**	**0.743**	**0.886**	**0.646**	0.199	**0.777**
my friendships and social life	**.784**	-.024	**0.747**	.597	.041	**.380**	**0.716**	**0.852**	**0.889**	-0.143	**0.761**
sex life	**.843**	-.232	**0.668**	-.139	-.243	**.786**	**0.588**	**0.847**	**0.809**	-0.12	**0.700**
physical appearance	**.761**	.130	**0.825**	.114	-.501	**.458**	**0.817**	**0.949**	**0.712**	0.208	**0.848**
things I could do physically	**.815**	.083	**0.847**	.190	-.294	**.572**	**0.777**	**0.848**	**0.758**	0.130	**0.835**
holidays or leisure activities	**.869**	-.070	**0.799**	**.930**	-.005	-.068	**0.756**	**0.901**	**0.933**	-0.126	**0.817**
ease of travelling	**.693**	.125	**0.757**	**.882**	-.036	-.016	**0.774**	**0.860**	**0.782**	0.032	**0.786**
my confidence in my ability to do things	**.815**	.063	**0.834**	**.793**	-.162	.103	**0.882**	**0.903**	**0.841**	0.050	**0.857**
motivation to achieve things	**.874**	-.010	**0.844**	**.836**	-.143	.051	**0.869**	**0.876**	**0.853**	0.032	**0.856**
Society reaction	**.889**	-.070	**0.819**	**.820**	-.183	-.157	**0.757**	**0.904**	**0.901**	-0.069	**0.828**
worries about the future	**.554**	.354	**0.771**	.311	**-.583**	.103	**0.831**	**0.757**	**0.513**	0.375	**0.777**
finances	**.766**	.159	**0.849**	.332	**-.629**	.110	**0.892**	**0.833**	**0.665**	0.272	**0.85**
need to depend on others	**.689**	-.128	**0.587**	.419	**-.543**	.017	**0.833**	**0.832**	**0.672**	0.046	**0.689**
living conditions	**.618**	.329	**0.817**	.261	**-.841**	-.161	**0.842**	**0.786**	**0.544**	0.379	**0.811**
freedom to eat	.172	**.867**	**0.735**	-.135	**-.961**	.147	**0.801**	**0.838**	0.132	**0.865**	**0.769**
enjoyment of food	.031	**.937**	**0.644**	-.156	**-.985**	.107	**0.779**	**0.851**	0.019	**0.943**	**0.716**
freedom to drink	-.025	**.941**	**0.593**	.105	**-.864**	-.017	**0.82**	**0.728**	-.021	**0.916**	**0.657**
Eigenvalue	10.404	1.897	**10.404**	11.229	1.9	1.12	**11.229**	**13.05**	11.056	1.459	**11.056**
Variance explained	57.801	10.537	**57.801**	62.382	10.556	6.223	**62.382**	**72.5**	61.422	8.605	**61.422**
Reliability	**0.955**			**0.963**				**0.977**	**0.962**		

**Fig 1:**
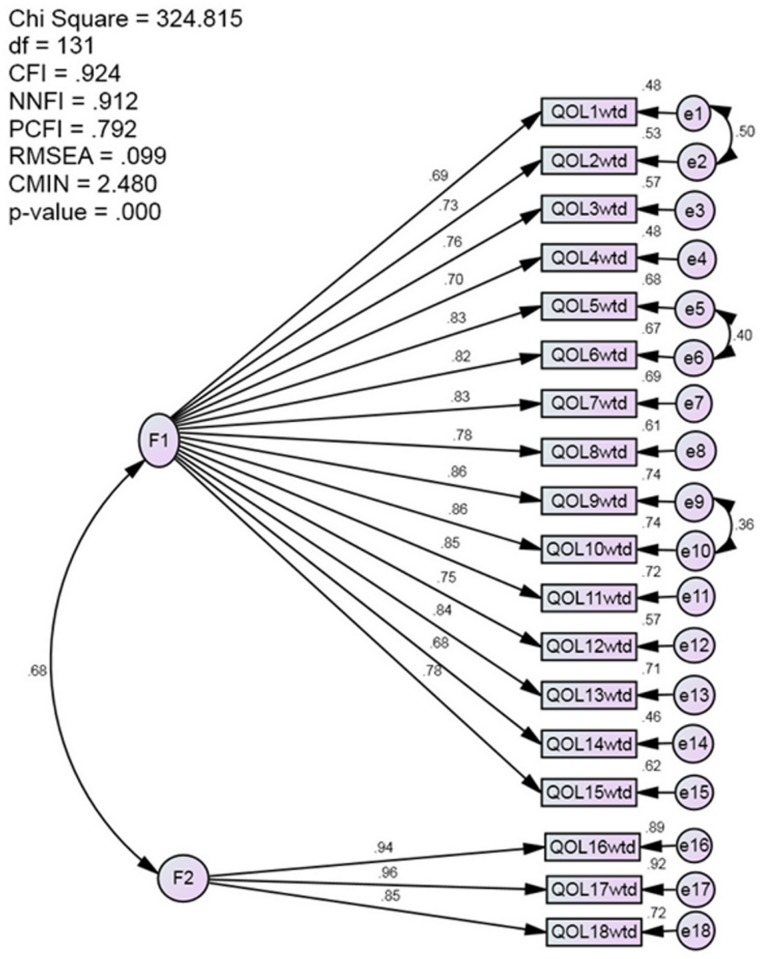
Two-factor model CFA. Satisfactory fit statistics that support two factors model.

**Fig 2:**
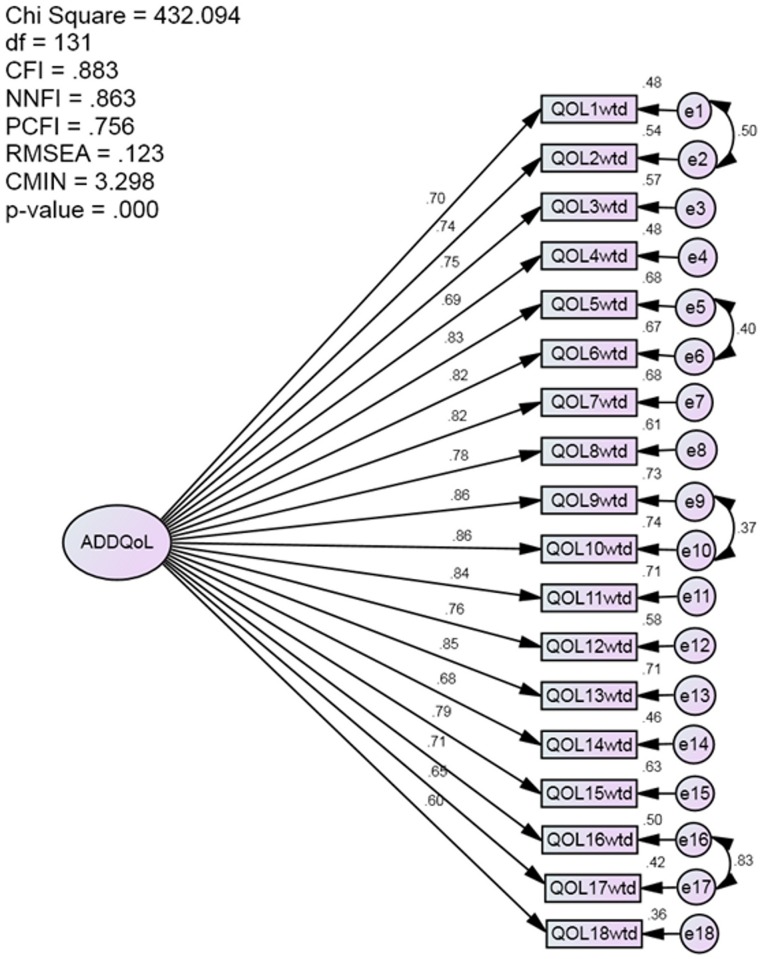
One factor model CFA. Fit statistics didn’t support one factor model.

CFA showed that two factors model has better fit statistics than one factor model (Figs 1 and 2); For two factors model, the first factor included the first 15 life domains found by EFA, the second factor included the three domains of freedom to eat, enjoyment of food and freedom to drink. Average Variance Explained (AVE) and Composite Reliability (CR) for first factors was (AVE = 0.619, CR = 960) and for second factor (AVE = 0.845 CR = 0.942). AVE for one factor model was 0.709 and CR = 0.978.

### Impact of diabetes on life domains

Table 3 shows the unweighted and weighted impact of DM on each life domain arranged from the highest to lowest impact. It is noted that diabetes mellitus affected freedom to eat and food enjoyment the most with negative impact of -2.04 (± 0.907) and -2.01 (± 0.91) respectively. The least domain found to be affected by the disease process is society reaction, which registered a mean and SD of -1.16 (± 1.20). A similar distribution with more negative impact was observed when the impact of DM was weighted by the perceived importance of life domains. Freedom to eat registered the highest negative impact -5.09 (± 3.03) and the lowest is “society reaction”, which registered a mean (± SD) of -2.95 (± 3.19). The rank of six domain’s mean had minimally changed after weighting.

**Table 3:** Impact of Diabetes on life domains and rank of unweighted and weighted domains.

Life Domain		Unweighted		Weighted	
	N	Mean(SD)	Rank	Mean(SD)	Rank
Freedom to eat	246	-2.04 (0.91)	1	-5.09 (3.03)	1
Enjoyment of food	247	-2.01 (0.935)	2	-5.06 (3.03)	2
Living conditions	246	-1.88 (0.96)	3	-4.73 (3.06)	3
Freedom to drink	247	-1.82 (1.15)	4	-4.65 (3.31)	4
Family life	228	-1.79 (0.91)	5	-4.48 (2.83)	5
Physical appearance	248	-1.78 (0.92)	**6**	-4.37 (2.79)	**7**
Things I could do physically	248	-1.77 (0.97)	**7**	-4.4 (2.94)	**6**
Confidence to do things	245	-1.73 (0.97)	8	-4.27 (2.93)	8
Working life	209	-1.70 (0.91)	9	-4.19 (2.72)	9
Motivation to achieve things	247	-1.64 (1.01)	**10**	-3.98 (2.86)	**11**
Worries about the future	246	-1.62 (1.13)	**11**	-4.02 (3.22)	**10**
Sex life	181	-1.51 (1.09)	12	-3.87 (3.12)	12
Ease of travelling	245	-1.49 (1.08)	13	-3.62 (3.08)	13
Holidays or leisure activities	244	-1.44 (1.06)	14	-3.48 (2.96)	14
Friendships and social life	249	-1.35 (1.07)	**15**	-3.25 (2.99)	**16**
Finances	246	-1.33 (1.12)	**16**	-3.41 (3.26)	**15**
Dependence on others	246	-1.32 (1.23)	17	-3.2 (3.52)	17
Society reaction	246	-1.16 (1.2)	18	-2.96 (3.19)	18

### Factors associated with QoL

Table 4 shows the results of hierarchical multiple linear regression. It is observable that none of the sociodemographic variables were associated with QoL. BMI, blood pressure, ischemic heart disease, nephropathy and foot ulcer were not associated with QoL. Patients with diabetes who had a desired glycaemic control had a more negative impact of diabetes on QoL. Longer duration of diabetes, being worried about complications, having neuropathy, and having retinopathy were associated with greater negative impact of diabetes on QoL. Being satisfied with waiting time in the clinic was associated with less negative impact of diabetes.

**Table 4:** Results of Hierarchical Linear Regression. Variables entered in * First block, †Second block, §Third block, ‡Fourth block.

Variables	B *(95%CI)*	t	P
Age in years*	-0.01(-0.031, 0.02)	- 0.47	0.636
Female gender*	0.00 (-0.46, 0.47)	0.02	0.985
Malay race*	0.25 (-0.44, 0.93)	0.71	0.477
Above secondary Education *	0.06 (-0.23, 0.34)	0.39	0.698
Religion other than Muslim*	-0.08 (-0.70, 0.54)	- 0.26	0.796
Married *	0.37(-0.02, 0.75)	1.9	0.059
Perception of a healthy status†	0.53 (0.16, 0.90)	1.69	0.093
Last Systolic Blood pressure†	0.00 (-0.01, 0.01)	0.45	0.655
Last Diastolic Blood pressure†	0.00 (-0.02, 0.03)	0.63	0.532
BMI†	-0.02 (-0.06, 0.03)	- 0.74	0.461
Having Ischemic Heart Disease †	0.02 (-0.76, 0.78)	0.04	0.965
Having renal complication †	-0.47 (-1.02, 0.08)	-1.69	0.093
Having Cardiovascual accident †	-0.92 (-2.18, 0.35)	-1.43	0.153
Having Foot ulcer †	-0.13 (-0.89, 0.63)	- 0.34	0.734
Achieved Glycaemic control†	-0.67 (-1.15,-0.19)	- 2.06	0.041
Being worried about diabetes †	-1.27 (-1.96,-0.66)	- 3.56	<0.001
More than 10 years disease duration†	-1.06 (-1.53,-0.58)	- 4.24	<0.001
Having Neuropathy †	-0.82 (-1.29,-0.34)	- 3.01	<0.001
Having Retinopathy †	-0.97 (-1.44,-0.49)	- 4.01	<0.001
Satisfied with waiting time†	0.94 (0.44,1.44)	3.49	0.001
Insulin Treatment§	-3.05 (-3.92, -2.18)	- 6.92	<0.001
Treatment*Glycaemic control‡	1.43 (0.420, 2.43)	2.8	0.006

A significant moderating effect of the treatment modality was observed, evident by the significant regression coefficient of treatment (p<0.001) and the significant interaction term (p = 0.006).

## Discussion

South East Asian countries are known for their diverse culture and life style. Obesity and DM are expected to be high among the population of this region in the coming 20 years [22]. Malaysia is a developing country that has achieved appreciable progress in the development of its economy, human capital, infrastructure and industry. The rapid socio-financial changes, acquisition of modern automation and diverse investments resulted in increased gross national product per capita, improved living conditions, and increasing accessibility to processed foods and other goods accompanied by increased life expectancy. Prevalence of diabetes has doubled in the last two decades; a prevalence of 15.2% in 2011 is more than double that of 1996 (6.3%) [6]. Countries with a Malay speaking population also reported a high prevalence of DM. The Singaporean prevalence of DM in 2010 (11%) is double of that in 1980s (5%)[23]. The affluent Malay country of Brunei is incurring an excess of 12.5% prevalence of DM [24]. Nonetheless, a lower prevalence was noticed in Indonesia (5.8%) [25]. The availability of a robust measurement tool would enable comparison across countries and identify factors that may affect QoL, especially those related to culture. Thus, precise measure of disease outcomes would help health professionals to design appropriate intervention(s) to improve QoL among an increasing number of patients with diabetes.

The distribution of respondents’ characteristics is in line with most of the published articles that included the three races of Malaysia, the majority of whom were Malays, followed by Chinese and Indians [26]. The age exhibited wide range with high mean. This finding was not unexpected as type 2 DM is a disease of adults. The distribution of educational level, employment status was expected not to conform to the Malaysian standards [26] as the sample is diabetic patients (i.e. a group who is seeking medical attention from general hospitals are assumed to be with lower employment and education).

The forced one factor solution has been supported for the whole sample and for each race indicating that the questionnaire is stable among different races in Malaysia and can be used for comparison in a Malaysian context irrespective of ethnicity. The high loadings with high internal consistency support one factor solution; none of the variables showed a problem in the loading or could affect reliability. The unforced factor structure which yielded a factor includes eating related items (life domains) is similar to the findings of other studies [11,13–15]. Nonetheless, CFA has showed that two factors model has better fit statistics compared to one factor model. The high correlation between the factors of ADDQoL allowed summation of these subscales into one total score in par with other scales where subscales are summed into single total score [27–28]. This might warrant the tendency to force the items to load into one factor as it is the case in all studies that tested the factor structure of ADDQoL.

It might be argued that the minor differences found are attributed to differences in culture. If this was the case, we would have seen “unforced one factor solution” of versions which were used in other countries, including the original version from the UK. Moreover, other experiences showed that only the forced one factor solution yielded the desired factor structure that allows summation of all weighted domains into single AWI score that reflects the impact of DM on QoL. The importance rating was evenly distributed between important and very important. Thus, multiplying the impact by rating did not change the ranking of domains substantially, which implied that participants had almost similar perceptions of life domains’ importance.

One aspect that merits discussion is that none of the sociodemographic variables were associated with AWI in this study. This reflects the sensitivity and specificity of ADDQoL that solely measures DM irrespective of sociodemographic factors. Age was not associated with diabetes-related QOL in this study, which goes along the findings of others studies [12–13] and opposes others [29–31]. As referred to previously, DM is typically a disease of adults; those groups may share similar characteristics in regard to mental or physical health. A difference would be expected when comparing a young age group to an older one who are assumed to be less physically active, prone to mental problems and having comorbidities. The role of ethnicity in QoL would be expected when some of the groups are impoverished, or have lower employment or less education [32]. Nonetheless, such attribution was not evident in our study due to cultural similarities regardless of age, sex and race in terms of food and lifestyle.

Obesity is a well known major risk factor for the development of diabetes and it was found to be correlated independently with QOL among diabetic and non diabetic patients[33–34]. Obese diabetic patients incur a double burden of disease. However in this study and similar studies where ADQQOL was used as a measurement tool, obesity (BMI) did not influence diabetes –related QOL [35]. The likely explanation is that detecting impact of obesity on QOL might need a special measurement tool as the effect of obesity has been elucidated with generic measurement tools. Moreover, the significant association between sociodemographic factors and QoL was mainly elucidated through generic health questionnaires, which usually involve physical mental or/and social domains. These questionnaires are incriminated as being a measurement of health status rather than a QoL[36]. In regards to factors associated with QoL, the finding that approximately 39.8% of respondents had had diabetes for more than 10 years, and 67.5% did not achieve the desired glycaemic control, may explain many of the observed results. Among those with long duration of diabetes, many complications are likely to have developed by time of assessment, adding another burden to these individuals. The results of this study are in line with many studies from different parts of the world where patients with diabetes complications reported worse QoL [11–12,17,30,37].

Similarly, glycaemic control tends to be worse with longer diabetes duration due to a decline in beta cell function, as well as a decline in patients’ attitude and adherence to treatment regimen. Published reports support the role the patient and health care provider might play in glycaemic control. It has been shown that maintaining a strict dietary regimen, punctuality on appointment and adherence to medication regimen would improve glycaemic control [38]. Interestingly, the patients with desired glycaemic control showed a more negative impact of diabetes on QoL than those with less than optimum control; this association was moderated by treatment modality (use of insulin). Patients who aimed to achieve desired glycaemic control are subjected to a strict management and life style regimen that was reportedly found to negatively affect QoL. Other studies also reported a negative impact of insulin use on QoL [8,11–12,39]. The results of this study contradict findings from other studies where those with desired glycaemic control had better QoL [40–42]. The positive impact on QoL of these studies is mainly attributed to the reduction of acute and long-term complications and improvement in physical health; glycaemic control reduces fear of complications, enhances self confidence in diabetes management and strengthens social interaction which would contribute to better mental and physical health[43]. Moreover, some studies showed that the negative impact of insulin therapy is usually offset by a positive effect of glycaemic control [44–46].

We have identified few limitations that might affect the interpretation. First, although the case mix was selected from a referral centre in the Capital of Malaysia, generalization of the results might have been affected by the inclusion of hospital-based patients who may not necessarily be representative of patients treated at health clinics. Secondly, a cross-sectional study does not measure within-person changes in QoL and may reflect historical changes in education and care provided: a longitudinal study is required to measure within-patient change over time. Thirdly, CFA couldn’t be done for each race individually because of the limited sample size.

## Conclusions

Our findings signify that the Malay ADDQoL-18 is a valid tool to be used among patients with diabetes from different ethnicities in Malaysia. Diabetes negatively impacted quality of life. The use of insulin to achieve better glycaemic control had more negative impact on QoL than the use of tablets and/or diet.

## References

[1] International Diabetes Federation (2013) Diabetes Atlas Brussels, Belgium: International diabetes federation.

[2] Whiting DR, Guariguata L, Weil C, Shaw J (2011) IDF Diabetes Atlas: Global estimates of the prevalence of diabetes for 2011 and 2030. Diabetes Research and Clinical Practice 94: 311–321. 10.1016/j.diabres.2011.10.029 22079683

[3] Al-Habori M, Al-Mamari M, Al-Meeri A (2004) Type II Diabetes Mellitus and impaired glucose tolerance in Yemen: prevalence, associated metabolic changes and risk factors. Diabetes Research and Clinical Practice 65: 275–281. 10.1016/j.diabres.2004.02.001 15331208

[4] Centers for Disease Control Prevention (2014) National diabetes statistics report: estimates of diabetes and its burden in the United States, 2014. Atlanta, GA: US Department of Health and Human Services.

[5] International Diabetes Federation (2014) Diabetes Atlas Brussels, Belgium: International diabetes federation.

[6] Institute for Public Health (2011) National Health and Morbidity Survey 2011 (NHMS 2011). Vol. 1: Methodology and General Findings.

[7] Letchuman G, Wan Nazaimoon W, Wan Mohamad W, Chandran L, Tee G, Jamaiyah H, et al (2010) Prevalence of Diabetes in the Malaysian National Health Morbidity Survey III 2006. Med J Malaysia 65: 173–179. 21939164

[8] Rubin RR, Peyrot M (1999) Quality of life and diabetes. Diabetes/Metabolism Research and Reviews 15: 205–218. 10441043 10.1002/(sici)1520-7560(199905/06)15:3<205::aid-dmrr29>3.0.co;2-o

[9] Watkins K, Connell CM (2004) Measurement of health-related QOL in diabetes mellitus. Pharmacoeconomics 22: 1109–1126. 10.2165/00019053-200422170-00002 15612830

[10] Bowden A, Fox-Rushby JA (2003) A systematic and critical review of the process of translation and adaptation of generic health-related quality of life measures in Africa, Asia, Eastern Europe, the Middle East, South America. Social Science and Medicine 57: 1289–1306. 10.1016/S0277-9536(02)00503-8 12899911

[11] Bradley C, Todd C, Gorton T, Symonds E, Martin A, Plowright R (1999) The development of an individualized questionnaire measure of perceived impact of diabetes on quality of life: the ADDQoL. Quality of Life Research 8: 79–91. 10.1023/A:1026485130100 10457741

[12] Da Costa FA, Guerreiro JP, Duggan C (2006) An audit of diabetes dependent quality of life (ADDQoL) for Portugal: exploring validity and reliability. Pharmacy Practice 4: 123–128. 25214898 PMC4156844

[13] Kamarul Imran M, Ismail A, Naing L, Wan Mohamad W (2007) The reliability and validity of the Malay version of the 18-item audit of Diabetes Dependent Quality of Life (the Malay ADDQOL) questionnaire. Southeast Asian Journal of Tropical Medicine and Public Health 38: 398–405. 17539293

[14] Ostini R, Dower J, Donald M (2012) The Audit of Diabetes-Dependent Quality of Life 19 (ADDQoL): feasibility, reliability and validity in a population-based sample of Australian adults. Quality of Life Research 21: 1471–1477. 10.1007/s11136-011-0043-0 22012024

[15] Wee H-L, Tan C-E, Goh S-Y, Li S-C (2006) Usefulness of the Audit of Diabetes-Dependent Quality-of-Life (ADDQoL) questionnaire in patients with diabetes in a multi-ethnic Asian country. Pharmacoeconomics 24: 673–682. 10.2165/00019053-200624070-00006 16802843

[16] Holmanová E, Žiaková K (2009) Audit diabetes-dependent quality of life questionnaire: usefulness in diabetes self-management education in the Slovak population. Journal of Clinical Nursing 18: 1276–1286. 10.1111/j.1365-2702.2008.02602.x 19077012

[17] Speight J, Sinclair A, Browne J, Woodcock A, Bradley C (2013) Assessing the impact of diabetes on the quality of life of older adults living in a care home: validation of the ADDQoL Senior. Diabetic Medicine 30: 74–80. 10.1111/j.1464-5491.2012.03748.x 22804615

[18] Quah CK (1999) Issues in the translation of English affixes into Malay. Journal des traducteurs 44: 604–616. 10.7202/003881ar

[19] Kaiser HF (1974) An index of factorial simplicity Psychometrika 39: 31–36. 10.1007/BF02291575

[20] Snell SA, Dean JW (1992) Integrated manufacturing and human resource management: A human capital perspective. Academy of Management Journal 35: 467–504. 10.2307/256484

[21] Cohen J, Cohen P, West SG, Aiken LS (2013) Applied multiple regression/correlation analysis for the behavioral sciences: Routledge.

[22] Jayawardena R, Ranasinghe P, Byrne NM, Soares MJ, Katulanda P, Hills AP (2012) Prevalence and trends of the diabetes epidemic in South Asia: a systematic review and meta-analysis. BMC Public Health 12: 380 10.1186/1471-2458-12-380 22630043 PMC3447674

[23] Phan TP, Alkema L, Tai ES, Tan KH, Yang Q, Lim W-Y, et al (2014) Forecasting the burden of type 2 diabetes in Singapore using a demographic epidemiological model of Singapore. BMJ open diabetes research & care 2: e000012 10.1136/bmjdrc-2013-000012 25452860 PMC4212579

[24] WHO (2012) Country and regional data on diabetes.

[25] International Diabetes Federation (2011) Diabetes Atlas Brussels, Belgium: International diabetes federation.

[26] Department of Statistics (2011) Population Distribution and Basic Demographic Characteristics 2010. Malaysia.

[27] McHorney CA, Ware JE Jr, Raczek AE (1993) The MOS 36-Item Short-Form Health Survey (SF-36): II. Psychometric and clinical tests of validity in measuring physical and mental health constructs. Medical Care: 247–263. 10.1097/00005650-199303000-00006 8450681

[28] Henry JD, Crawford JR (2005) The short-form version of the Depression Anxiety Stress Scales (DASS-21): Construct validity and normative data in a large non-clinical sample. British Journal of Clinical Psychology 44: 227–239. 10.1348/014466505X29657 16004657

[29] Eljedi A, Mikolajczyk RT, Kraemer A, Laaser U (2006) Health-related quality of life in diabetic patients and controls without diabetes in refugee camps in the Gaza strip: a cross-sectional study. BMC Public Health 6: 268 10.1186/1471-2458-6-268 17074088 PMC1634853

[30] Papadopoulos AA, Kontodimopoulos N, Frydas A, Ikonomakis E, Niakas D (2007) Predictors of health-related quality of life in type II diabetic patients in Greece. BMC Public Health 7: 186 10.1186/1471-2458-7-186 17663782 PMC1973072

[31] Shiu AT, Thompson DR, Wong RY (2008) Quality of life and its predictors among Hong Kong Chinese patients with diabetes. Journal of Clinical Nursing 17: 125–132. 10.1111/j.1365-2702.2007.02036.x 18298763

[32] Quandt SA, Graham CN, Bell RA, Snively BM, Golden SL, Stafford JM, et al (2007) Ethnic disparities in health-related quality of life among older rural adults with diabetes. Ethnicity and Disease 17: 471 17985500 PMC2621317

[33] Banegas JR, López-García E, Graciani A, Guallar-Castillón P, Gutierrez-Fisac JL, Alonso J, et al (2007) Relationship between obesity, hypertension and diabetes, and health-related quality of life among the elderly. European Journal of Cardiovascular Prevention & Rehabilitation 14: 456–462. 10.1097/HJR.0b013e3280803f29 17568249

[34] Fontaine K, Barofsky I (2001) Obesity and health-related quality of life. Obesity reviews 2: 173–182. 10.1046/j.1467-789x.2001.00032.x 12120102

[35] Kolotkin RL, Crosby RD, Williams GR (2003) Assessing weight-related quality of life in obese persons with type 2 diabetes. Diabetes Research and Clinical Practice 61: 125–132. 10.1016/S0168-8227(03)00113-X 12951281

[36] Bradley C (2001) Importance of differentiating health status from quality of life. The Lancet 357: 7–8. 10.1016/S0140-6736(00)03562-5 11197385

[37] Issa B, Baiyewu O (2006) Quality of life of patients with diabetes mellitus in a Nigerian Teaching Hospital. Hong Kong Journal of Psychiatry 16: 27.

[38] Rhee MK, Slocum W, Ziemer DC, Culler SD, Cook CB, El-Kebbi IM, et al (2005) Patient adherence improves glycemic control. The Diabetes Educ 31: 240–250. 10.1177/0145721705274927 15797853

[39] Wexler D, Grant R, Wittenberg E, Bosch J, Cagliero E, Delahanty L, et al (2006) Correlates of health-related quality of life in type 2 diabetes. Diab tologia 49: 1489–1497. 10.1007/s00125-006-0249-9 16752167

[40] Akinci F, Yildirim A, Gözü H, Sargın H, Orbay E, Sargın M (2008) Assessment of health-related quality of life (HRQoL) of patients with type 2 diabetes in Turkey. Diabetes research and clinical practice 79: 117–123. 10.1016/j.diabres.2007.07.003 17707943

[41] Fischer JS, McLaughlin T, Loza L, Beauchamp R, Schwartz S, Kipnes M (2004) The impact of insulin glargine on clinical and humanistic outcomes in patients uncontrolled on other insulin and oral agents: an office-based naturalistic study. Current Medical Research and Opinion 20: 1703–1710. 10.1185/030079904X5526 15537471

[42] Houlden R, Ross S, Harris S, Yale J-F, Sauriol L, Gerstein HC (2007) Treatment satisfaction and quality of life using an early insulinization strategy with insulin glargine compared to an adjusted oral therapy in the management of Type 2 diabetes: the Canadian INSIGHT Study. Diabetes Research and Clinical Practice 78: 254–258. 10.1016/j.diabres.2007.03.021 17490781

[43] Lau C-Y, Qureshi A, Scott S (2004) Association between glycaemic control and quality of life in diabetes mellitus. Journal of Postgraduate Medicine 50: 189 15377803

[44] Diabetes Control Complications Trial Research Group (1996) Influence of intensive diabetes treatment on quality-of-life outcomes in the diabetes control and complications trial. Diabetes Care 19: 195–203. 10.2337/diacare.19.3.195 8742561

[45] De Grauw W, Van de Lisdonk E, Van Gerwen W, Van den Hoogen H, Van Weel C (2001) Insulin therapy in poorly controlled type 2 diabetic patients: does it affect quality of life? British Journal of General Practice 51: 527–532. 11462311 PMC1314043

[46] Pibernik-Okanović M, Szabo S, Metelko Ž (1998) Quality of life following a change in therapy for diabetes mellitus. Pharmacoeconomics 14: 201–207. 10.2165/00019053-199814020-00008 10186460

